# In Search of Relevant Urinary Biomarkers for Thyroid Papillary Carcinoma and Benign Thyroid Nodule Differentiation, Targeting Metabolic Profiles and Pathways via UHPLC-QTOF-ESI^+^-MS Analysis

**DOI:** 10.3390/diagnostics14212421

**Published:** 2024-10-30

**Authors:** Gabriela Maria Berinde, Andreea Iulia Socaciu, Mihai Adrian Socaciu, Gabriel Emil Petre, Armand Gabriel Rajnoveanu, Maria Barsan, Carmen Socaciu, Doina Piciu

**Affiliations:** 1 Department of Occupational Health, University of Medicine and Pharmacy “Iuliu Hatieganu”, Str. Victor Babes 8, 400347 Cluj-Napoca, Romania; gabriela.berinde@umfcluj.ro (G.M.B.); armand.rajnoveanu@umfcluj.ro (A.G.R.); maria.opritoiu@umfcluj.ro (M.B.); 2 Department of Medical Imaging, University of Medicine and Pharmacy “Iuliu Hatieganu”, Str. Victor Babes 8, 400347 Cluj-Napoca, Romania; mihai.socaciu@umfcluj.ro; 3 Department of Surgery 4, University of Medicine and Pharmacy “Iuliu Hatieganu”, Str. Victor Babes 8, 400347 Cluj-Napoca, Romania; dr_gabipetre@yahoo.com; 4 Research Center for Applied Biotechnology and Molecular Therapy BIODIATECH, SC Proplanta SRL, Str. Trifoiului 12G, 400478 Cluj-Napoca, Romania; csocaciu@proplanta.ro; 5 Doctoral School, University of Medicine and Pharmacy “Iuliu Hatieganu”, Str. Victor Babes 8, 400347 Cluj-Napoca, Romania; doina.piciu@gmail.com

**Keywords:** papillary thyroid cancer, benign nodular goiter, urinary metabolites, metabolic pathways, thyroid putative biomarkers

## Abstract

Background: Identification of specific urine metabolic profiles for patients diagnosed with papillary thyroid carcinoma (TC) vs. benign nodules (B) to identify specific biomarkers and altered pathways compared to those of healthy controls (C). Methods: Patient urine samples were collected, before surgery and after a histological confirmation of TC (*n* = 30) and B (*n* = 30), in parallel with sample collection from healthy controls (*n* = 20). The untargeted and semi-targeted metabolomic protocols were applied using UPLC-QTOF-ESI^+^-MS analysis, and the statistical analysis was performed using the Metaboanalyst 6.0 platform. The results for the blood biomarkers, previously published, were compared with the data obtained from urine sampling using the Venny algorithm and multivariate statistics. Results: Partial least squares discrimination, including VIP values, random forest graphs, and heatmaps (*p* < 0.05), together with biomarker analysis (AUROC ranking) and pathway analysis, suggested a specific model for the urinary metabolic profile and pathway alterations in TC and B vs. C, based on 190 identified metabolites in urine that were compared with the serum metabolites. By semi-targeted metabolomics, 10 classes of metabolites, considered putative biomarkers, were found to be responsible for specific alterations in the metabolic pathways, from polar molecules to lipids. Specific biomarkers for discrimination were identified in each class of metabolites that were either upregulated or downregulated when compared to those of the controls. Conclusions: The lipidomic window was the most relevant for identifying biomarkers related to thyroid cancer and benign conditions, since this study detected a stronger involvement of lipids and selenium-related molecules for metabolic discrimination.

## 1. Introduction

Thyroid diseases significantly affect the entire metabolic balance, as well as the emotional and social life of humans. Different pathologies can be distinguished, expressed by inflammation (thyroiditis), hyperthyroidism, hypothyroidism associated with benign thyroid nodules, or thyroid carcinoma [1,2,3,4]. TC is a frequent endocrine tumor with a growing incidence worldwide, exhibiting a complex epidemiology, including different environmental and occupational risk factors and lifestyle influences. Benign nodules frequently induce development of a goiter, an enlargement of the thyroid gland, mainly associated with hypothyroidism or a normal hormone level (euthyroidism). Papillary thyroid carcinoma (TC) is the most common of all thyroid carcinomas and accounts for more than 80% of malignant endocrine tumors and about 65% of malignant thyroid cancers. According to the new WHO classification of thyroid tumors and recently reviewed advances in the diagnosis and therapy of thyroid cancer [5,6], thyroid cancer is ranked as the 9th leading cancer in the GLOBOCAN 2020 database [7]. It is frequently diagnosed in adults (30–50 years old), especially in women (three times more often than in men), with a good prognosis for early diagnosis [8]. Despite research efforts, current knowledge of the etiology of TC remains limited; up to 50% of the new cases in the thyroid carcinoma group are papillary microcarcinomas, with nodules measuring less than 1 cm.

TC is typically detected via an ultrasound-guided fine-needle aspiration biopsy and cytological examination of the specimen after surgery [9]. This approach has significant limitations due to the small sample size and the inability to characterize follicular lesions adequately. According to a recent review, emerging biomarkers, such as mRNA and non-coding RNAs, can potentially detect thyroid neoplasms in clinical settings, e.g., the miRNA, lncRNA, and circRNA dysregulation in several thyroid neoplasms, recommending their potential to act as diagnostic and prognostic biomarkers [10]. Also, different protein biomarkers that are able to differentiate benign and malignant thyroid nodules are currently used, as recently reviewed [11]. Although the use of needle aspiration can reduce unnecessary thyroid surgery, the prevalence of non-diagnostic and indeterminate histopathology is still high, and the costs of mutation tests, including nucleic acid and protein-based diagnosis markers of differentiated thyroid carcinomas, are high [11].

Clinically, the diagnosis of thyroid hormone dysfunction is primarily based on biochemical indicators, such as serum thyroid stimulating hormone (TSH) and serum free thyroxine (FT4) levels, often used to diagnose thyroid disorders or to monitor treatment response; however, whether or not these parameters fully capture TH status remains controversial. The diagnostic criteria for hypothyroidism include serum TSH levels above the upper reference limit and FT4 levels within or below the reference range. An emerging high-throughput technology can aid in this diagnosis by correlating certain serum metabolomic phenotypes with the metabolic status and outcomes of endocrine diseases [12,13]. It is acknowledged that over time, as reviewed recently, TH affects many aspects of the lipid metabolism, from synthesis, to mobilization, to degradation. It increases the activity of 3-hydroxy-3-methyl-glutaryl coenzyme A reductase, cholesteryl ester transfer protein hepatic lipase, and the lecithin–cholesterol ratio.

Circulating biomarkers help in understanding the TC metabolism and may provide an early diagnosis via a minimally invasive technique, since tumor cells are metabolically overactive and undergo significant metabolic reprogramming to sustain their proliferation. The tumor tissue consumes large amounts of glucose through glycolysis (Warburg effect); therefore, the glucose-related metabolites are substantially altered in TC. Amino acids, as a resource for protein synthesis, are involved in metabolic reprogramming and can promote tumor proliferation and metastasis; therefore, disturbances in the metabolism of some amino acids have also been observed in TC, although the effects remain unclear. Lipids and their metabolites involved in cell membrane formation, signaling, and energy storage are associated with carcinogenic pathways. Lipid metabolism is reprogrammed in tumors, and the perturbation of blood lipids has been identified as a risk factor for tumorigenesis; therefore, they represent an important feature of TC and provide diagnostic biomarkers [14]. Both subclinical and overt hypothyroidism were also associated with alterations in the lipid profile, particularly proportional to TSH upregulation, correlated with the increase in LDL cholesterol and an increased risk of adverse metabolic and cardiovascular outcomes [15].

Until now, as reviewed recently, no reliable specific molecular markers for the detection and staging of TC have been standardized, the assessment of metabolic changes in the development of thyroid benign (B) nodules vs. TC being limited to the measurement of individual hormones (mainly TSH) and some metabolite levels using standard clinical laboratory tests.

In this context, new, reliable, and affordable biomarkers for early detection of different thyroid pathologies are needed to complete and improve current methods. The “omics” technologies address distinct directions of diagnosis and investigation, from genomics to proteomics and metabolomics. Diverse techniques applied for an accurate molecular diagnosis includes genomic and proteomic analyses, as well as tissue imaging by MALDI/Fourier transform/mass spectrometry or by AFADESI-MSI (mass spectrometry imaging), presenting a spatially resolved metabolic profiling of endogenous metabolites in thyroid tumors by the identification of metabolites in situ on a tissue slice obtained by fine-needle aspiration biopsy [16]. The serum/urine metabolic fingerprint and profile can identify the potential biomarkers using advanced techniques like high-performance gas- or liquid chromatography coupled with mass spectrometry (GC/UPLC-MS) or NMR. Metabolomics is an emerging technology which can separate, identify, and classify different classes of small molecules (<5000 Da), including intracellular or extracellular metabolites, signaling mediators, nutrients, that reflect the “downstream” of altered metabolic pathways responsible for benign or malignant transformation, having an important impact in clinical and translational research [17,18,19]. Some metabolic alterations in TC were identified by techniques based on GC-MS and UPLC-MS or NMR, which are increasingly used for metabolite separation and identification [6,7,20,21,22,23,24,25,26,27].

In the last decade, a growing interest in finding specific metabolite alterations in tissue samples in PTC patients and discriminating the profiles against those for benign nodules has been reported. Tissue oncometabolites, e.g., higher lactate and choline levels and low levels of citrate, together with glutamine and glutamate, were considered putative biomarkers for nodules, while cholesterol, choline, and phosphocholines displayed significantly different values in TC patients than in healthy subjects. Through MS imaging analysis of seven TC cases, the distribution of phosphatidylcholines (16:0/18:1 16:0/18:2) and sphingomyelin (d18:0/16:1) compared to that in normal tissues was significantly higher in TC [22]. The results for three tissue metabolites involved in the galactose metabolism pathway were defined as a combinatorial biomarker affecting the energetic metabolism, assisting in the needle biopsy for TC diagnosis, as demonstrated by the receiver operating characteristic (ROC curve and AUC value of 0.96). Therefore, Alpha-galactosidase (GLA) is a potential target for TC therapy [28]

As mentioned previously, in recent years, relevant progress has been made in thyroid pathology using tissue and blood metabolomics. Very few data regarding urine metabolites are available [29] except for those from a recent review concerning the general status of urine sampling used as a liquid biopsy for noninvasive cancer research, including its preanalytical parameters and protocols from health sciences databases [30]. Our previous research reviewed the updated literature regarding TC diagnostics related to occupational and environmental risk factors [31] and published original data regarding the serum metabolic biomarkers which may discriminate between TC and B patients [32]. Herein, this study aimed to perform a metabolomic analysis of urine samples collected simultaneously with blood samples from the same patients diagnosed with TC and B in order to identify specific biomarkers in each of these samples (1), as well as to compare the specific metabolic profiles in serum vs. urine, identifying the common vs. specific biomarkers of discrimination when compared to those of healthy subjects (2). The advanced metabolomics analysis, coupled with multivariate statistics, was performed using the Metaboanalyst 6.0 standardized procedures, also identifying specific metabolic pathways responsible for the alteration of metabolic pathways in benign vs. malign thyroid pathology (3) and providing a reliable metabolic analysis of the thyroid pathophysiology in urine vs. serum.

## 2. Materials and Methods

### 2.1. Patients and Study Design

This study complied with the guidelines of the Declaration of Helsinki and the Conference for Coordination of Clinical Practice and was approved by the Ethics Committee for Scientific Research (DEP224/26 July 2022) of the Iuliu Hatieganu University of Medicine and Pharmacy, Cluj-Napoca, Romania. Written informed consent was obtained from all 80 subjects. A total number of 20 healthy subjects (group C) and 60 patients diagnosed with different thyroid pathologies, i.e., papillary carcinoma and microcarcinoma, were included in the TC group (*n* = 30), and patients with a nodular goiter, confirmed to have clinical hypothyroidism, were included in the B group (*n* = 30), as shown in Table 1. The urine samples (together with blood samples) were collected between 2020–2022 from a University Hospital in Cluj-Napoca, Romania. Here, we present the data regarding the urine samples.

The data for the inclusion/exclusion criteria and for the analysis of the risk factors were collected from the patients’ clinical history and pathology reports. We collected information regarding the type of thyroid disease, with TNM staging, for TC patients, along with data regarding associated diseases in the personal medical history, demographic data, family history, previous medication, lifestyle (tobacco smoking, alcohol consumption, nutritional status), and environmental and occupational risk factors.

The inclusion criteria consisted of the following: history of thyroid pathology or no previous history of thyroid pathology, using the diagnostic criteria for thyroid diseases and histological classification; clear consciousness; no intellectual impairment; and normal communication, as presented in Table 1.

The exclusion criteria included the following: patients that refused to participate in this study, pregnant or lactating women, those with combined malignancy, patients with psychiatric disorders, and those with incomplete data for the diagnostic criteria or inconclusive pathology findings.

### 2.2. Urine Collection and Pre-Analytical Procedures

Urine samples were collected from the first morning urine, before surgery, in sterile vials and were preserved, after the addition of 0.1% Na-azide, in a deep freezer. A volume of 0.8 mL pure HPLC-grade (Merck KGaA, Darmstadt, Germany) Methanol and Acetonitrile (2:1 *v*/*v*) was added for each volume of 0.2 mL cold urine. The mixture was vortexed to precipitate proteins, ultrasonicated for 5 min, and stored 24 h at −20 °C to increase protein precipitation. After centrifugation at 12.500 rpm for 10 min (4 °C), the supernatant was collected, filtered through nylon filters (0.2 μm), and introduced into glass micro vials before being injected into the LC-MS system. The ultra-high-performance liquid chromatograph (UHPLC) quality control (QC) samples were also prepared at the same time; 10 μL were collected from each sample and added to 2 ml Eppendorf (Eppendorf Corporation, Hamburg, Germany) microtubes, vortexed, and divided into 0.2 mL portions for each tube and then pretreated using the same procedure to improve the data quality for metabolic profiling.

### 2.3. UHPLC-QTOF-ESI^+^-MS Analysis

The metabolomic profiling was performed using UHPLC coupled with electrospray ionization quadrupole time-of-flight mass spectrometry (UHPLC-QTOF-ESI^+^-MS) using a Thermo Fisher Scientific (Waltham, MA, USA) UHPLC Ultimate 3000 instrument equipped with a quaternary pump, a Dionex delivery system, and MS detection equipment with MaXis Impact (Bruker Daltonics, Billerica, MA, USA). The metabolites were separated on an Acclaim C18 column (5 μm, 2.1 × 100 mm, pore size of 30 nm) (Thermo Fischer Scientific, Waltham, MA USA) at 28 °C. The mobile phase consisted of 0.1% formic acid in water (A) and 0.1% formic acid in acetonitrile (B) (LiChrosolv^®^ MerckMillipore, Burlington, MA, USA). The gradient program and the MS parameters were detailed previously [32]. The control of the instrument and the data processing was performed using TofControl 3.2, HyStar 3.2, Data Analysis 4.2 (Bruker, Daltonics, Billerica, MA, USA), and Chromeleon software 7.3, respectively.

### 2.4. Statistical Analysis

Subsequent to the sampling using UHPLC-QTOF-ESI^+^-MS Analysis, up to 429 molecules were identified. For each sample, the raw data consisted of base peak chromatograms (BPC), representing the intensity of each molecule vs. the retention time (min). Then, a matrix cumulating all samples was obtained, for which the step-by-step methodology applied was presented previously [32]. A final number of 190 molecules were identified and considered for multivariate and univariate analysis using the Metaboanalyst 6.0 platform (https://www.metaboanalyst.ca, accessed on 26 September 2024). The identification of each molecule was performed using the Human Metabolome Database (HMDB) and the LipidMaps platforms. The experimental *m*/*z* values representing [M+H]^+^ (M—molecular weight of the molecule) in the ESI^+^ system of fragmentation were compared with the average of the theoretical *m*/*z* values found in the International Database HMDB, with the theoretical accuracy related to the experimental *m*/*z* values being below 20 ppm.

The multivariate analysis of the detected molecules identified in all samples was conducted via a partial least squares discriminant analysis (PLSDA) and random forest (RF)-based prediction test and illustrated by heatmap clusters. The values of variable importance in the projection (VIP), as well as mean decrease accuracy (MDA) by RF analysis, were calculated, and the ranking of the most significant molecules explaining the discrimination was achieved. According to the biomarker analysis algorithm, the ROC curves were obtained, and the AUROC values (area under the curve) were considered as the best prediction of differentiation for the putative biomarkers. The pathway analysis was also applied, based on the identified cohort of 190 molecules. To identify common and specific molecules in urine and blood, the Venny 2.1 algorithm was applied (https://csbg.cnb.csic.es, accessed on 26 September 2024).

## 3. Results

### 3.1. Untargeted Metabolic Profiles

**Figure 1:**
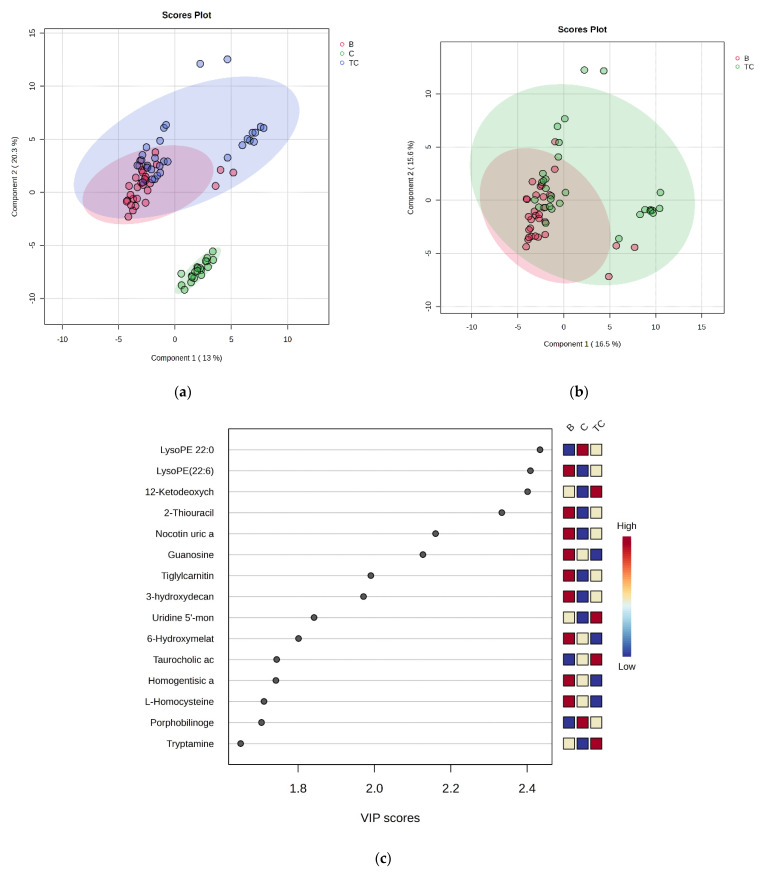
(**a**) PLSDA score plots discriminating groups TC, B, and C. (**b**) PLSDA score plots for groups TC vs. B. (**c**) The top 15 molecules discriminating between the three groups of samples, with VIP scores >1.6.

According to the raw data obtained by the UHPLC-TOF-ESI^+^-MS analytics and raw data processing, matrices containing *m*/*z* values and peak intensities of 190 metabolites belonging to 10 classes of metabolites were selected and identified, as presented in Table S1 (Supplementary File T1). According to the PLSDA score plots, the discrimination regions between groups C vs. TC vs. B were determined, with a covariance of 33.3% (Figure 1a). The cross-validation analysis showed a good accuracy (0.8), with R2 values > 0.6 and Q2 values > 0.5 for the first three components, confirming an acceptable predictability and reliability of the model. When comparing the TC vs. B samples, the covariance was 32.1% (Figure 1b), but we did not identify any significant differences between these groups, according to the cross-validation analysis.

The VIP scores >1.6 revealed the first 15 molecules to be considered significant for the discrimination between the TC and B groups (Figure 1c). The most significant differences were noticed for guanosine, 6-hydroxymelatonin, homogentisic acid, homocysteine (with lower levels in the TC group compared with those in the B group), and taurocholic acid (with higher levels in the TC group). These data were correlated with complementary statistics, as shown below.

**Figure 2:**
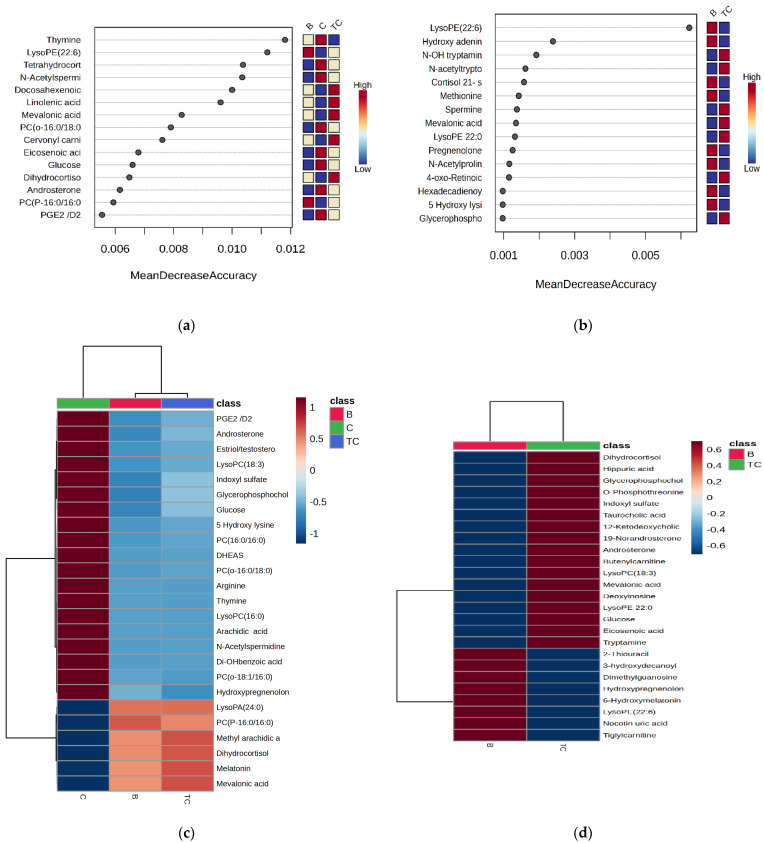
MDA values, according to RF analysis (**a**,**b**) and heatmaps (**c**,**d**), used to differentiate the metabolomic profiles of thyroid cancer (TC) samples compared to the profiles for group C (control) and group B, respectively.

Random forest analysis is a tool used for the suitable classification of putative biomarkers that we used for discriminating between groups C vs. TC vs. B and TC vs. B, respectively (Figure 2a,b). Heatmaps illustrate the relative abundance of molecules in each group by color intensity, showing increased or decreased levels of molecules, based on mean peak intensities (Figure 2c,d)

**Figure 3:**
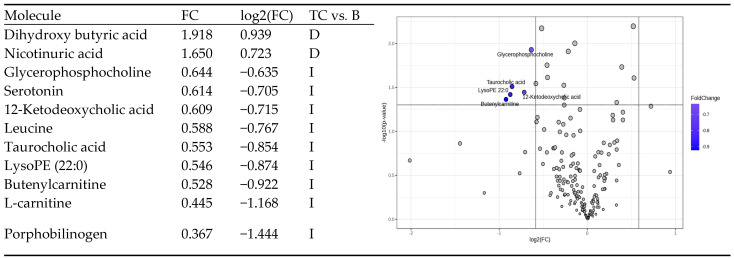
Volcano plot, FC, and log2(FC) values showing the molecules with significant decreases (D) or increases (I) in group TC vs. group B.

The MDA values higher than 0.006 (Figure 2a) showed putative biomarkers for differentiation between groups C, TC, and B. Lower values were noticed for docosahexaenoic, linolenic, and mevalonic acids in the TC group. When comparing the TC vs. B groups, the MDA values were below 0.0006 (Figure 2b), and increased levels of hydroxy tryptamine and N-acetyl tryptophan were identified for the TC group compared to those for the B group. The heatmaps illustrate 25 molecules identified with decreased (19) or increased (6) levels in the TC and B groups compared to the results for the controls. In general, most of these molecules displayed lower levels in the B and TC groups compared to those in the C group (Figure 2c). When comparing the TC vs. B groups, 17 molecules showed increased levels, and 8 molecules displayed decreased levels in the TC group (*p* < 0.05) (Figure 2d). Applying the volcano plot analysis, the fold-change values and log2 (FC) showed a semi-quantitative evaluation of the most significant differences between groups TC and B (Figure 3).

Significant decreases were noticed for dihydroxy butyric acid and nicotinuric acid in group TC, while significant increases in glycerophosphocholine, serotonin, 12-Ketodeoxycholic acid, Leucine, Taurocholic acid, LysoPE 22:0, and Butenyl carnitine were detected in this group when compared to those of the B group.

### 3.2. Common and Specific Molecules Found in Urine Comparative to Blood Serum

Considering the molecules identified in urine (*n* = 190) and in serum (*n* = 166), according to our previous data [32], the Venny 2.1 algorithm was applied, identifying 90 common and 100 specific molecules in urine and 76 in serum. The complete list of these molecules is presented in Supplementary File T2.

### 3.3. Pathway Analysis

**Figure 4:**
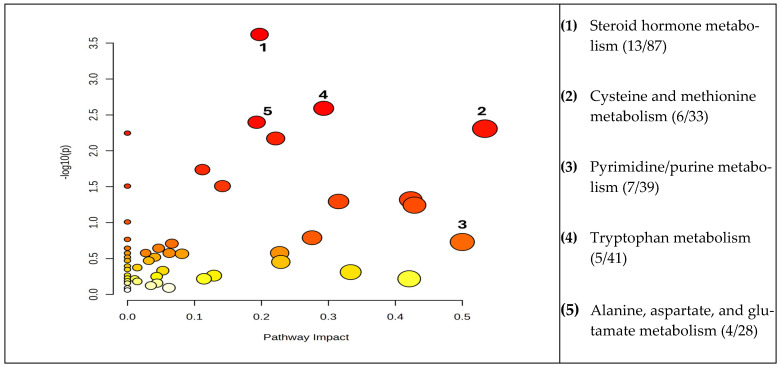
Specific metabolic pathways in urine as seen in thyroid pathology (TC and B), based on the results obtained in this study.

The cohort of molecules separated and identified in all groups was subjected to pathway analysis, considering the matched metabolic pathways from the pathway enrichment analysis and the impact values (*p* values < 0.05). We also compared the data obtained from urine with previous data obtained from blood samples [32]. A higher value on the y-axis (−log10p) indicates a lower *p*-value (threshold), while the x-axis gives the pathway impact value. The dimensions and color of the circles present in Figure 4 illustrate the impact for the first five metabolic pathways in urine.

In urine, the alteration of steroid amino acid metabolism had the highest impact, followed by pyrimidine/purine and amino acid metabolism. As reported previously [32], in blood, the order of impact (from 1 to 0.323) was Phenylalanine, tyrosine, and tryptophan biosynthesis > Linoleic/linolenic acid metabolism > Glutamine and glutamate metabolism > Tryptophan metabolism > Pyrimidine/purine metabolism. In general, the impact of lipid metabolism, especially steroid derivative values, was more pronounced in the urine compared to the blood samples.

### 3.4. Biomarker Analysis

Using Metaboanalyst 5.0, the biomarker analysis was applied to obtain the ROC curves and AUROC values for diagnostic purposes, considering the biomarkers’ sensitivity vs. specificity. The metabolites with the highest AUROC values were good biomarker candidates, useful for differentiating between groups TC and B in urine; additionally, a comparative analysis using blood serum samples results was conducted [32]. Table 2 includes these data.

The AUROC values for the top 15 molecules ranged from 0.730 to 0.645, as shown in Table 2. According to these data, in urine, mainly lipid molecules, such as LysoPE 22:6 and LysoPE 22:0), Mevalonic acid, Dihydrocortisol, Glycerophosphocholine, Androsterone, and 19-Norandrosterone, showed a higher sensitivity vs. specificity relevance, while in blood, the amino acid derivatives were more representative as biomarker candidates. Many of these molecules were also confirmed as potential biomarkers by complementary statistics in both urine and blood samples.

### 3.5. Semi-Targeted Approach and Statistics for Specific Metabolite Classes

**Figure 5:**
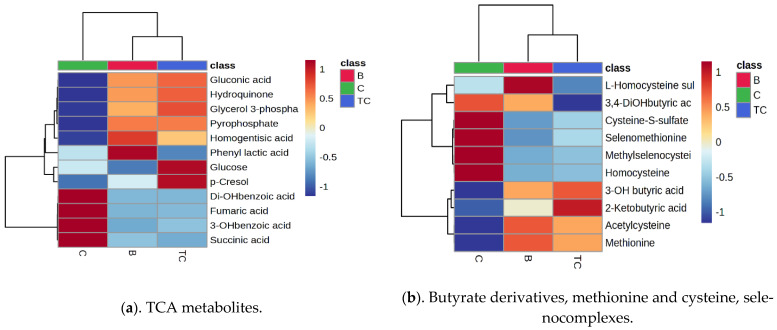

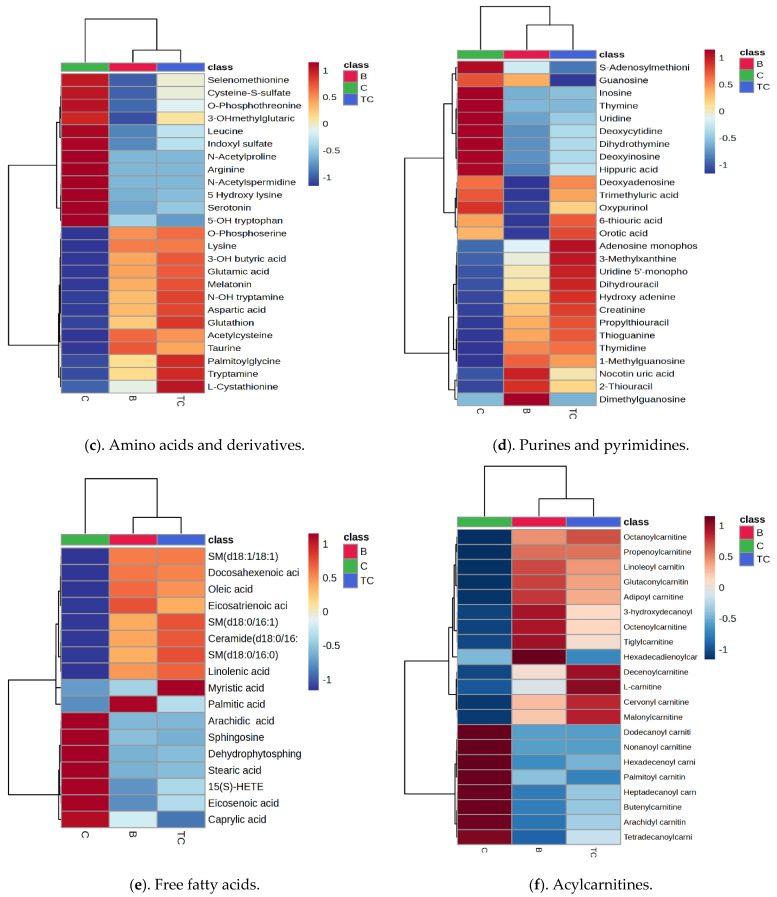

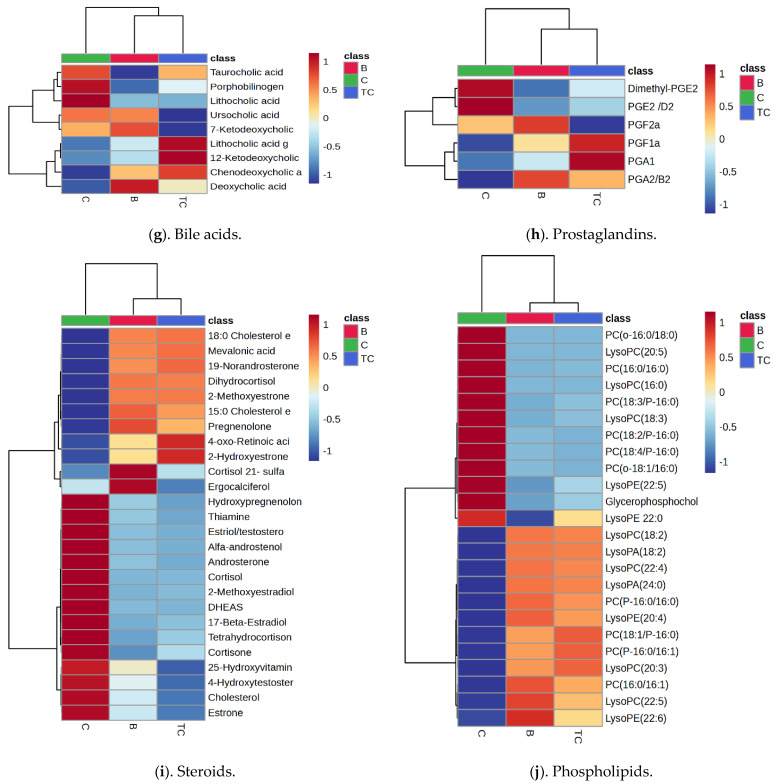
(**a**–**j**) Heatmaps corresponding to different classes of urine metabolites, which illustrate the differences between the TC, B, and C groups. (**a**) TCA metabolites. (**b**) Butyrate derivatives, methionine and cysteine, selenocomplexes. (**c**) Amino acids and derivatives. (**d**) Purines and pyrimidines. (**e**) Free fatty acids. (**f**) Acylcarnitines. (**g**) Bile acids. (**h**) Prostaglandins. (**i**) Steroids. (**j**) Phospholipids.

Ten different classes of metabolites involved in specific metabolic pathways were identified in urine. For each class, the matrices (*m*/*z* values vs. peak intensities) were analyzed using Metaboanalyst platform statistics in order to more specifically identify, for each class, the molecules which may discriminate between TC and B vs. C samples. Supplementary Files (S1 and S2) include VIP score graphs and RF graphs, respectively, corresponding to each class of metabolites and illustrating the differences between TC, B, and C groups. Figure 5a–j illustrates the heatmaps obtained for each class of metabolites by applying Ward’s clustering method, with Euclidian distance and an ANOVA *p*-value (FDR) cutoff of 0.05.

Regarding the polar metabolites and their related pathways (Figure 5a–d), the heatmaps illustrate either similar or specific variations for TC and B groups. Alterations in the TCA metabolism were observed by decreased levels of fumaric, succinic acids, hydroxy, and dihydroxy benzoic acid in both the TC and B groups; increased levels of phenyl lactic acid in group B; and increased release of glucose and p-cresol in the TC group (a). Therefore, the levels of phenyl lactic acid, glucose, and p-cresol can differentiate TC from B. Modifications in the release of the butyrate derivatives methionine and cysteine selenocomplexes due to lower levels of homocysteine and its sulfates, dihydroxy butyrate, selenomethionine, and methylselenocysteine, were noticed in both the TC and B groups. Specific increases in homocysteine sulfate in group B and ketobutyric acid in group TC were also observed (b). The alterations in the amino acid metabolism are represented by decreased leucine, arginine, acetyl proline, hydroxylysine, serotonin, and hydroxytryptophan in both the TC and B groups. Meanwhile, many other amino acid derivatives (from phosphoserine to cystathionine) can vary between the TC and B samples (c). Alterations in the nucleotide and nucleoside levels were also identified, with decreased levels specifically of guanosine, inosine, thymine, uridine, deoxycytidine, and hippuric acid. Guanosine and dimethyl guanosine seem to act as good markers to differentiate between the TC and B groups (d). Regarding the lipid metabolites (Figure 5e–j), the heatmaps illustrate more important differences between the TC and B groups, especially for bile acids and prostaglandins. The levels of some unsaturated free fatty acids were increased in both the TC and B groups, when compared to those for the controls, except for myristic and palmitic acid, which seems to differentiate group TC from B. The B group showed the highest levels of palmitic acid, while the TC group had the highest levels of oleic and linolenic acids (e). A large population of acylcarnitines known to be actively involved in the transport of acyl groups of fatty acids to the mitochondria was identified. Besides L-carnitine, twelve molecules showed increased levels in the TC vs. the B and controls groups, while six specimens exhibited increased levels only in the TC group (f). Bile acid metabolism was significantly affected, with increased release of keto deoxycholic, lithocholic, and chenodeoxycholic acids in group TC, significantly differing from the results for the B and control groups (g). The prostaglandins also showed a good discrimination between the three groups. The increased release of PGA1 and PGF1a satisfactorily differentiated the TC from the B group, as did decreased PGF2a levels in the TC group and increased levels in the B group (h). Steroids (corticosteroids, vitamins, and sex hormones) also exhibited altered levels in the TC and B groups compared to those in the control group. There were no significant differences between the TC and B groups, except in regards to estrone, hydroxy estrone, hydroxy testosterone, oxo-retinoic acid, 25 hydroxyvitamin D, and ergocalciferol levels (i). Phospholipids, especially LysoPC with different fatty acids and LysoPE 22:0, showed variations among the three groups, some with significant increases in the TC group, especially for the unsaturated fractions (j). These data are consistent with our results regarding pathway analysis and reveal details about the specific variations of different classes of metabolites, identifying either common metabolic signatures of the TC and B groups or specific alterations for each pathology group when compared to controls.

These findings agree with the previous LC-MS or NMR experimental data obtained in serum samples and confirm the specific alterations of these metabolic pathways [13,15,24,25,33,34,35,36,37]. Only one reference related to the urine metabolic profile compared to that of serum has been published previously in a similar study, “Biomarkers for TC, benign thyroid nodule and healthy controls”. Differentiation metabolites such as serum β-hydroxybutyrate, docosahexaenoic acid, 1-methyladenosine, pregnanediol-3-glucuronide, as well as nicotinuric acid mononucleotide and xanthosine, were validated to be significantly different, with AUROC values above 0.94, thus being considered suitable biomarkers for TC and potentially useful for clinical diagnosis [38]. Our findings reveal novel data and information, especially considering lipid metabolism.

## 4. Discussion

Over the last decade, systematic reviews regarding the potential of proteomic and metabolomic techniques to elucidate mechanisms involved in thyroid pathology, especially TC, have been published [16,22,24,25,26,39,40,41]. Tissue, blood serum, and plasma samples were mainly studied, with metabolic alterations of subclinical and clinical relevance identified in hypothyroidism and TC.

Serum TSH and FT4 levels are the major determinants of clinical hypothyroidism; however, the metabolic alterations compared to the levels in healthy controls remain poorly defined. Using untargeted UHPLC-MS plasma metabolomics, the influence of hypothyroidism on metabolism was recently investigated [13]. There were identified dysregulated pathways in clinical hypothyroidism compared to those of the controls, especially in regards to primary bile acid biosynthesis, steroid hormone biosynthesis, lysine degradation, tryptophan metabolism, and purine metabolism. Around 65 metabolites and five primary metabolism pathways were significantly associated with TSH and FT4 serum levels using machine learning algorithm prediction models. Few studies suggested a possible association between hypothyroidism and bile composition and excretion, e.g., FT4 stimulates hepatic BA synthesis and the biliary secretion of lipids (cholesterol and phospholipids), while TSH promotes cholesterol synthesis and inhibits BA synthesis. BAs act as signaling molecules regulating the thyroid pituitary axis and are associated with energy expenditure. Primary BAs, including cholic acid, taurocholic acid, and taurodeoxycholic acid, were found to be decreased in the serum of patients with hypothyroidism. Concomitantly, significantly higher levels of glycerophospholipids, including PC, PE, PI, PS, and Lyso (PC, PE, PA), were identified as potential biomarkers for the prediction of hypothyroidism. Steroid hormones (especially DHEA-S, androsterone glucuronide, and pregnanediol) were also decreased. The amino acid metabolism was notably altered, with reduced levels of L-tyrosine, L-lysine, and L-histidine. Also, the levels of the 5-hydroxytryptamine, tryptophan metabolism, and lysine degradation metabolic pathways were markedly altered compared to those of the controls. Tryptophan plays a crucial role in protein synthesis and is a precursor for the biosynthesis of 5-hydroxytryptamine, melatonin, kynurenic acid, and tryptamine, as well as the reduced secretion of tryptophan and kynurenine, contributing to hypothyroidism. The purine metabolism pathway was also significantly disturbed, with patients with hypothyroidism showing increased levels of purine metabolites (adenine, hypoxanthine, and xanthine).

The fact that the metabolism of tumor cells is altered has been known for many years, although the mechanisms and consequences of metabolic reprogramming have just begun to be understood. To satisfy tumor cell proliferation and survival requirements, the biosynthetic cell capabilities are increased, including the stimulation of fatty acid and nucleotide synthesis. Glucose is the substrate preferred by glycolysis for energy supply, providing pyruvate, ATP, and NADH, the electron donor in the mitochondria enabling the oxidative phosphorylation pathway, which yields much more ATP than glycolysis. Moreover, glucose provides metabolites used as building blocks for the biosynthesis of fatty acid and nucleotide biosynthesis, required for cellular proliferation. Acetyl-CoA enters the TCA cycle to supply energy for tumor cells and is needed for fatty acid biosynthesis, as well as for some amino acids to be converted to proteins. Therefore, the metabolic pathways are reprogrammed, and glycolysis is strongly enhanced to fulfil the high ATP demands of these cells. The interactions between the altered metabolic pathways (glutamate and glutamine metabolism and the TCA cycle) and lipid biosynthesis and metabolism in the plasma of TC patients were recently demonstrated, identifying some significant biomarkers such as sebacic acid, L-glutamine, and indole-3-carboxaldehyde [14]. In this context, similar metabolic alterations were found in TC and benign hypothyroidism when compared to those of the controls, but also including recently noticed specific features [36]. Based on tissue metabolomics, differences in the level of 3-hydroxybutyric acid (an intermediate of fatty acid metabolism) in the TC and control groups, as well as differences in carnitines ratios and sphingosine and sphingosine-1-phosphate levels, indicate that these molecules can be considered potential diagnostic biomarkers. The benign pathology was associated with an increased FFAs metabolism, while lower FFAs levels were associated with the increased consumption of lipids in TC. Increased bile acid levels were correlated with the upregulated fatty acid metabolism in the B group. Meanwhile, proline and glutamic acid derivatives levels were elevated, and the levels of hydroxybutyric and di-hydroxybutyric acids, intermediates of fatty acid metabolism, were lower in the TC vs. the B and control groups.

The main categories of metabolites considered as biomarker candidates for TC diagnosis were either polar molecules or lipids, such as estrogens, responsible for thyroid dysfunction [13,20,21,32,33,35]. The untargeted and targeted metabolomics revealed several tissue molecules, including carbohydrates (glucose, fructose, galactose, mannose, and rhamnose), 2-keto-gluconic and malonic acids, several amino acids, purine and pyrimidine metabolites, fatty acids, cholesterol and arachidonic acid, and choline-derived phospholipids. Citrate and lactate are the most significant biomarkers that can be used. Elevated tissue concentrations of some amino acids (methionine, leucine, tyrosine, and lysine), polar lipids (especially phospholipids and sphingolipids), together with the upregulated de novo synthesis of fatty acids in TC plasma, are also significant [39,40].

Recently, a combined metabolomic and lipidomic analysis of plasma samples from TC patients compared to healthy controls revealed 113 differential metabolites and 236 differential lipids, mainly involved in energetic and branched-chain amino acid metabolism, glutamate and glutamine metabolism, the tricarboxylic acid cycle, and lipid metabolism, providing new targets for comprehensive TC treatment [14].

In serum samples, increased levels of hydroxybutyric acid, docosahexaenoic, and 1-methyladenosine were noticed [35], as well as alterations of 42 metabolites, including proline betaine, and decreases in LysoPC (18:0 and 18:1) [16]. A large study performed on serum metabolomics for TC compared with control samples, identified proline betaine, taurocholic acid, L-phenylalanine, retinyl beta-glucuronide, alpha-tocotrienol, and threonine acid as being upregulated in the TC group, while L-tyrosine, L-tryptophan, 2-arachidonylglycerol, citric acid, and 42 other metabolites were downregulated. The Warburg effect was reflected by alterations in aspartic acid and glutamic acid metabolism, the urea cycle, and the tricarboxylic acid cycle, all involved in TC pathogenesis [21].

The metabolomic profile of patients with follicular tumors vs. benign nodules, analyzed by LC-MS in another study [25], identified six types of lipids, which may explain the differences between their metabolic profiles. These included amino acids (L-glutamate, L-glutamine), Lyso (PA, PC) derivatives of C16, C18, C20 and C22 fatty acids, sphingomyelin (d18:0/12:0), and linoleic acid. It was concluded that altered LysoPA levels may be one cause of follicular tumor carcinogenesis caused by a lipid metabolic pathway dysregulation.

Acyl-CoA is used in beta-oxidation inside the mitochondria or, depending on the mitochondrial status, is conjugated with L-carnitine to form acylcarnitines, which play an essential role in regulating the balance of intracellular carbohydrates and lipid metabolism. They serve as carriers to transport activated fatty acids into the mitochondria for β-oxidation, as a major source of energy for cell activities. Therefore, acylcarnitines are strongly involved in thyroid pathology, which is related to lipid metabolism. In our study, we observed a high concentration of urinary acylcarnitines, which correlates with the results of our previous study, in which we observed an increased serum level of acylcarnitines [32].

These findings were confirmed by our previous study focused on blood serum metabolomics [32], confirming the involvement of 10 classes of metabolites with specific metabolic pathways, which could differentiate patients from TC and B groups.

Selenium and its complexes with amino acids (methionine and cysteine), especially selenomethionine and selenocysteine, abundant in the thyroid tissue, play an essential role in thyroid hormone metabolism, although the specific mechanisms are not yet fully understood. Selenocysteine is in the active site of the three peroxidases, and its deficiency is currently a very common condition, having an inverse correlation with TC evolution, as documented by many authors [37,42,43,44]. Our data, in agreement with the results of our previous study [32], showed decreases in selenomethionine and methylselenocysteine levels in both the TC and B groups in comparison to those of the controls, in good agreement with the above-mentioned experimental findings.

There are several limitations of the current study. First, we included patients diagnosed with nodular goiter and clinical hypothyroidism in the B group, according to their TSH and FT4 values, without considering different comorbidities of hypothyroidism which might be seen in other clinical studies. Due to the exclusion criteria, the study displayed a relatively small sample size, targeting mainly differentiated thyroid cancer, specifically the papillary form; thus, our findings need to be further validated in larger scale studies.

## 5. Conclusions

Using untargeted metabolomics based on UHPLC-QTOF-ESI^+^-MS technology, urine samples from patients diagnosed with thyroid carcinoma and benign nodular goiters were compared with samples from healthy subjects. Specific urine putative biomarkers of discrimination were identified by complementary statistics provided by the integrated Metaboanalyst 6.0 platform and compared with the serum metabolic profiles obtained from the same patients. Ten different classes of metabolites, upregulated or downregulated in comparison to those of the controls, were targeted and compared, including a pathways analysis. The metabolomic window showed altered pathways for amino acid metabolism, carbohydrate-related TCA metabolites, selenium complexes, and purine and pyrimidine metabolites. Specific molecules were identified as putative biomarkers of differentiation, as detailed above. The lipidomic window proved to be more relevant for finding biomarkers related to thyroid carcinoma or benign nodules, such as alterations in free fatty acid metabolism, acylcarnitines and bile acids, steroid hormones, phospholipids, and prostaglandins.

The overall picture of the urinary metabolome, resulting from multivariate statistics complemented with biomarker and pathway analysis, was compared with a similar picture of the serum metabolome, finding both similar and remarkably different metabolites, both upregulated or downregulated, in thyroid carcinoma vs. benign nodules as compared with the levels for healthy controls.

## Figures and Tables

**Table 1:** Demographic and clinical data of subjects in groups TC, B, and C (control).

	Group PTC		Group B		Group C
Number of participants	30		30		20
Age in years (mean ± SD)	57.9 ± 4.3	56.6 ± 5.1	55.6 ± 4.8	57.9 ± 5.0	43± 5.5
Gender M/F	3/18	2/7	4/20	0/6	9/11
Body mass index (kg/m^2^)					
<30	8		11		16
≥30	22		19		4
Histological type	29 TC including microcarcinomas and 1 Medullary carcinoma		24 Nodular Goiter 2 Hashimoto’s 4 benign solitary nodules		0
TSH (mIU/L) (mean ± SD)	2.4 ± 0.62		17.9 ± 9.8		1.8 ± 0.7
FT3 (pmol/L) (mean ± SD)	5.9 ± 2.5		4.3 ± 2.6		5.2 ± 1.3
FT4 (pmol/L) (mean ± SD)	20.5 ± 6.60		11.2 ± 2.8		15.2 ± 5.8
First-degree relatives with thyroid or other cancer types					
Yes	6		3		3
No	24		27		17
Tobacco smoking (years of smoking cigarettes)					
No	4		5		14
<5	5		4		2
5–15	14		15		3
>15	7		6		1
Alcohol consumption (days/week)					
No	10/30		14/30		12/20
<5/week	12/30		10/30		6/20
>5/week	8/30		6/30		2/20

**Table 2:** Ranking of the 15 highest AUROC values for the putative biomarkers in the TC vs. B groups for both urine and serum. Log 2FC negative or positive values indicate the relative decrease (D) or increase (I), respectively, for metabolites identified in these groups.

TC vs. B (Urine)	AUROC	log2FC	TC vs. B (Blood Serum)	AUROC	Log2FC
LysoPE (22:6)	0.720	0.562	LysoPA (18:2)	0.730	−1.921
Mevalonic acid	0.699	−0.118	Taurine	0.718	0.444
Dihydrocortisol	0.692	−0.059	Acetylcysteine	0.716	0.343
Glycerophosphocholine	0.687	−0.517	LysoPE (20:4)	0.683	−1.155
O-Phosphothreonine	0.672	−0.348	Inosinic acid	0.677	−0.416
Hippuric acid	0.667	−0.428	L-Palmitoylcarnitine	0.676	−0.790
Androsterone	0.663	0.001	Cervonyl carnitine	0.676	−1.183
LysoPE 22:0	0.662	−0.810	Stearic acid (C18:0)	0.668	0.407
19-Norandrosterone	0.660	−0.162	Uridine 5′-diphosphate	0.661	−1.325
Porphobilinogen	0.656	−1.176	Arachidonic acid (C20:4)	0.660	−0.892
Tetrahydrocortisone	0.651	−0.452	PGE2 /D2	0.655	0.145
2-Thiouracil	0.649	0.608	5 Hydroxy tryptophan	0.651	0.156
Hydroquinone	0.648	−0.020	7-Methyl-cholic acid	0.649	0.864
LysoPC (18:3)	0.648	−0.358	LysoPC (20:4)	0.649	−1.011
Guanosine	0.647	0.347	Hypotaurine	0.645	0.319

## Data Availability

Data are contained within the article and Supplementary Materials.

## Supplementary Materials

The following supporting information can be downloaded at: https://www.mdpi.com/article/10.3390/diagnostics14212421/s1, Table S1. Urine molecules (*n* = 190) separated using HPLC-QTOF-ESI^+^-MS and identified according to their *m*/*z* values. The experimental *m*/*z* values were compared with the average of theoretical *m*/*z* values from the International Database HMDB (Human Metabolomic Database). Table S2. Common and specific molecules identified in urine vs. blood serum, as determined using the Venny 2.1 algorithm. Figure S1. VIP score graphs corresponding to different classes of metabolites, illustrating the differences between TC, B, and C groups. Figure S2. RF graphs (MDA values) corresponding to different classes of metabolites, illustrating the differences between TC, B, and C groups.
