# Supplementary material for: In Search of Relevant Urinary Biomarkers for Thyroid Papillary Carcinoma and Benign Thyroid Nodule Differentiation, Targeting Metabolic Profiles and Pathways via UHPLC-QTOF-ESI+-MS Analysis
Source: Diagnostics (Basel). 2024 Oct 30;14(21):2421. doi: 10.3390/diagnostics14212421 (PMC11544950; doi:10.3390/diagnostics14212421)
Supplement: Supplementary file 1 [file diagnostics-14-02421-s001.zip › Supl file Figure S1 VIP scores per classes .pdf]

Figure S1 VIP score graphs corresponding to different classes of metabolites, illustrating the differences between TC, B and C groups.

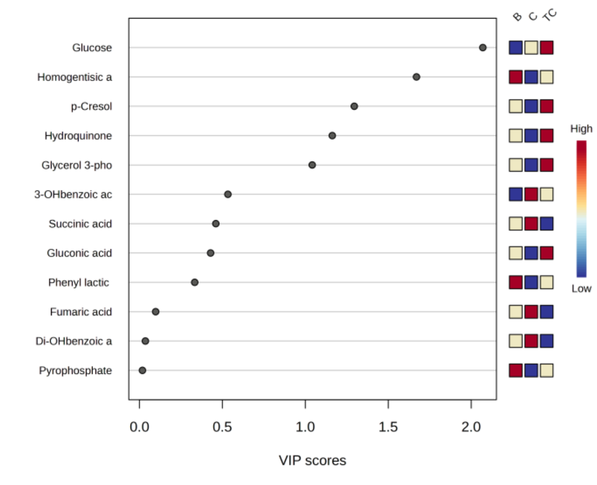

a. TCA metabolites

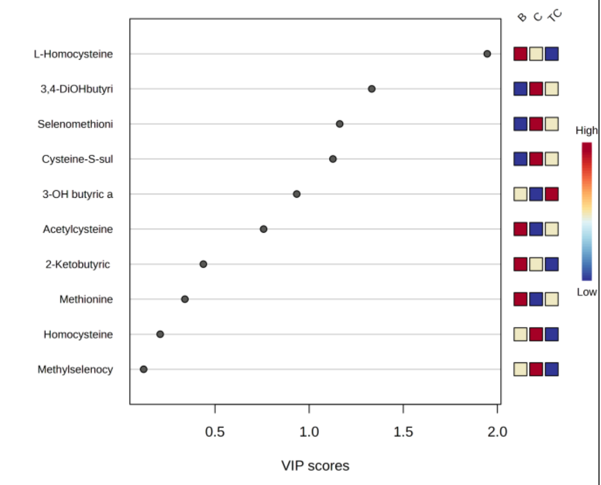

b. Butyrate derivatives, Methionine&Cysteine, Selenocomplexes

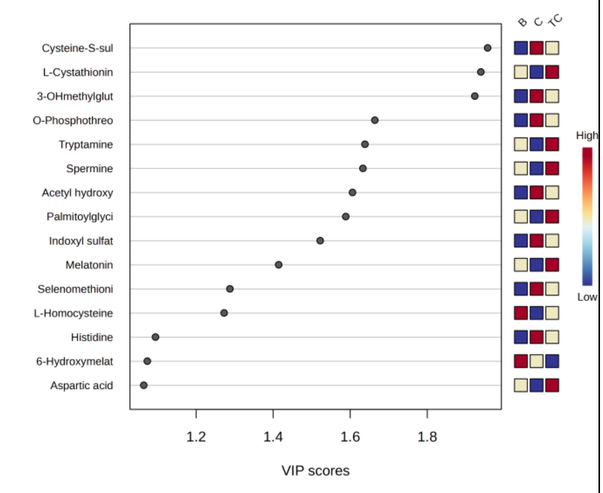

c. Amino acids and derivatives

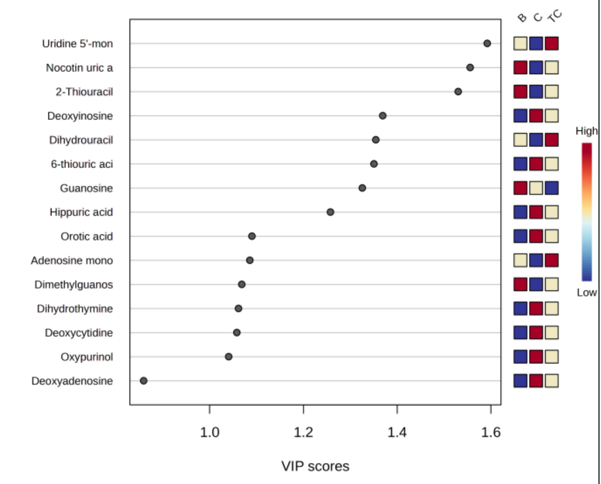

d. Purines and pirimidines

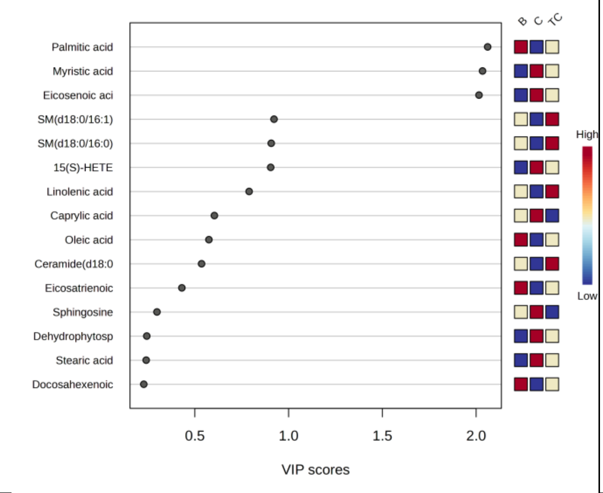

e. Free Fatty Acids

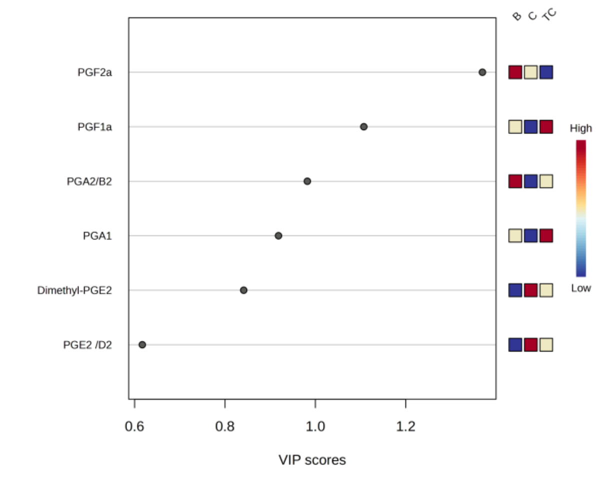

f. Acylcarnitines

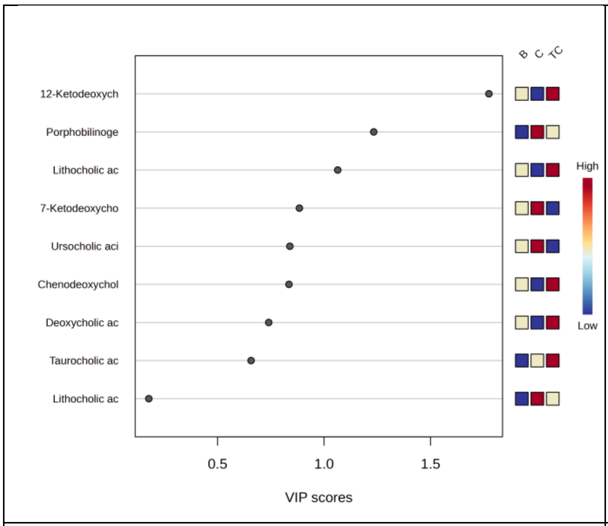

g. Bile acids

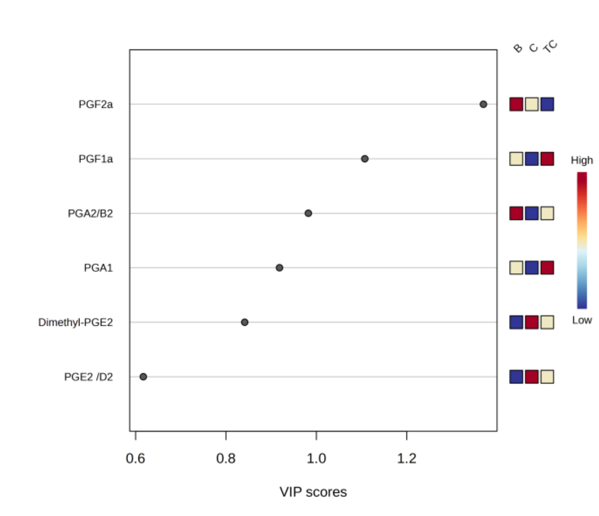

h. Prostaglandins

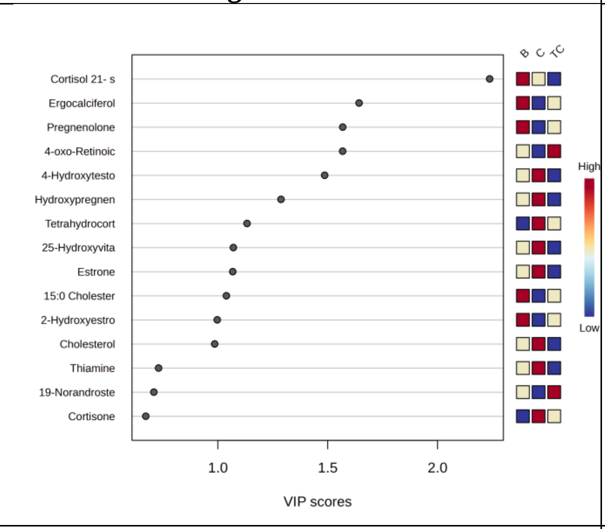

i. Steroids

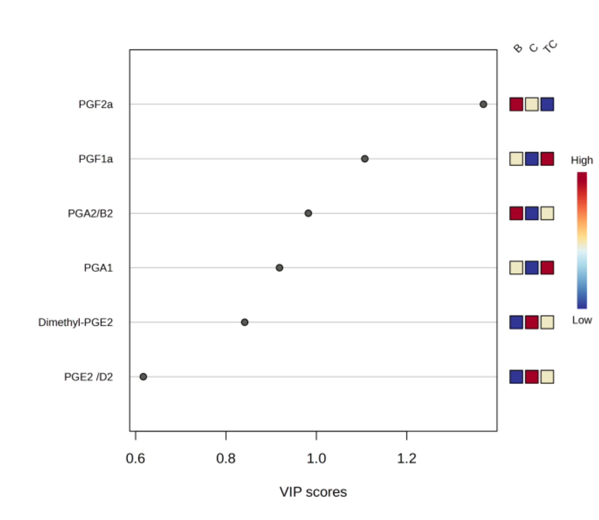

j. Phospholipids
