# Supplementary material for: In Search of Relevant Urinary Biomarkers for Thyroid Papillary Carcinoma and Benign Thyroid Nodule Differentiation, Targeting Metabolic Profiles and Pathways via UHPLC-QTOF-ESI+-MS Analysis
Source: Diagnostics (Basel). 2024 Oct 30;14(21):2421. doi: 10.3390/diagnostics14212421 (PMC11544950; doi:10.3390/diagnostics14212421)
Supplement: Supplementary file 1 [file diagnostics-14-02421-s001.zip › Supl file Figure S2 RF comparativ.pdf]

**Figure S2.** RF graphs (MDA values) graphs corresponding to different classes of metabolites, illustrating the differences between TC, B and C groups.

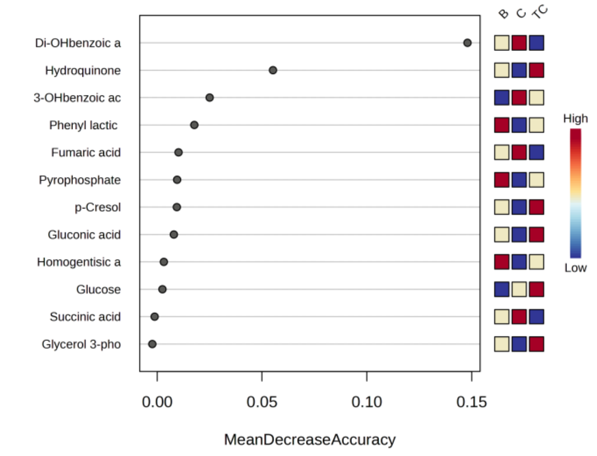


**a.** TCA metabolites

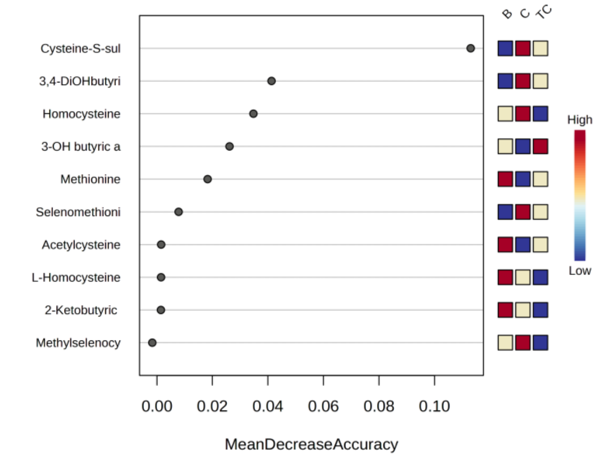


**b.** Butyrate derivatives, Methionine&Cysteine, Selenocomplexes

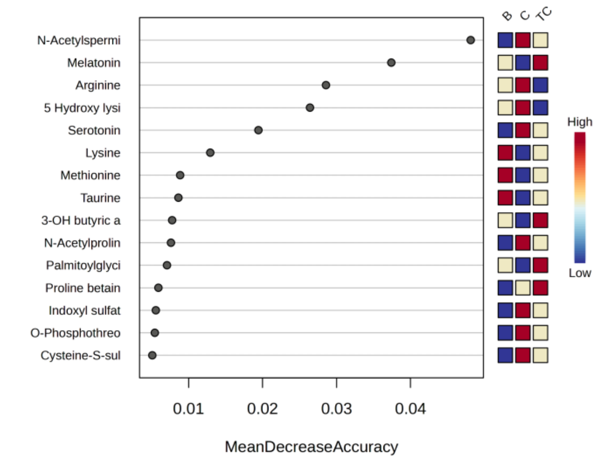


**c.** Amino acids and derivatives

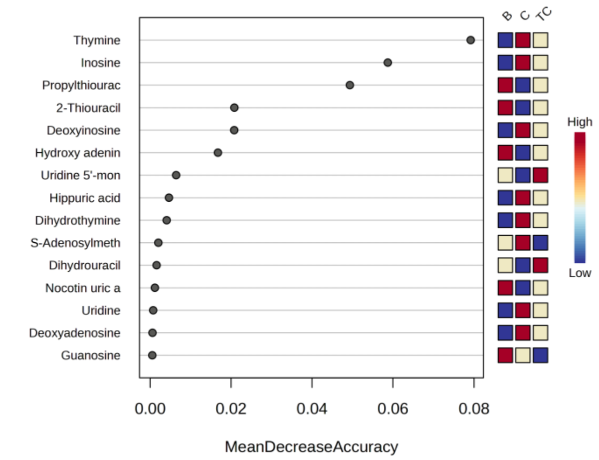


**d.** Purines and pirimidines

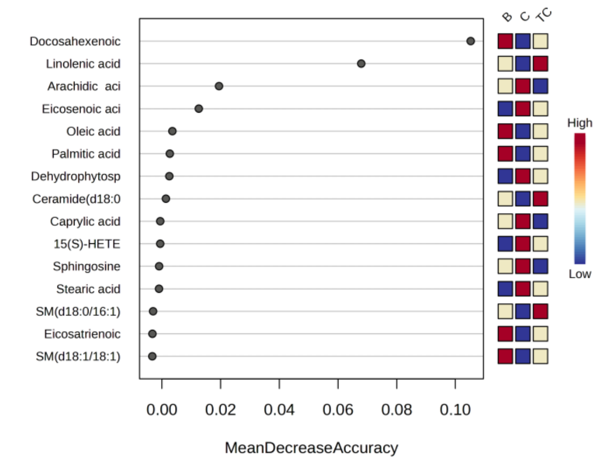


**e.** Free Fatty Acids

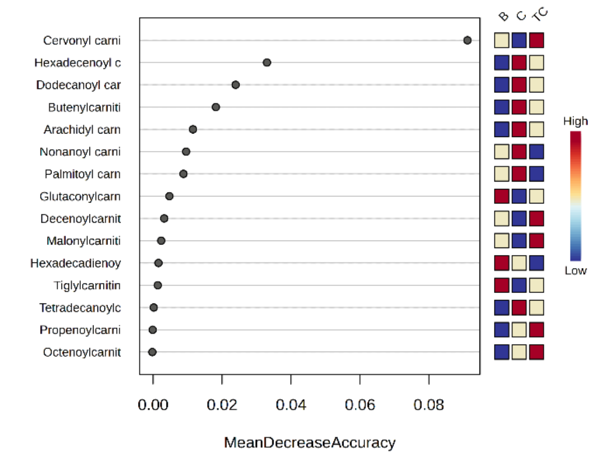


**f.** Acylcarnitines

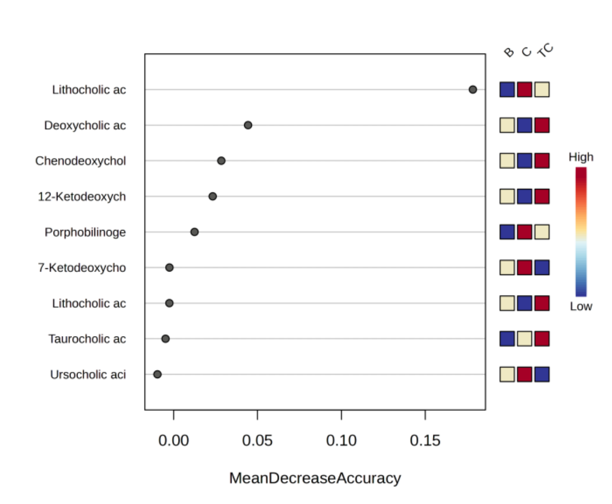


**g.** Bile acids

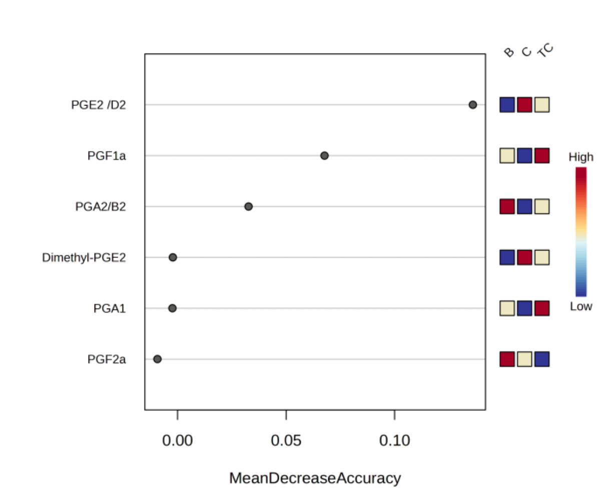


**h.** Prostaglandins

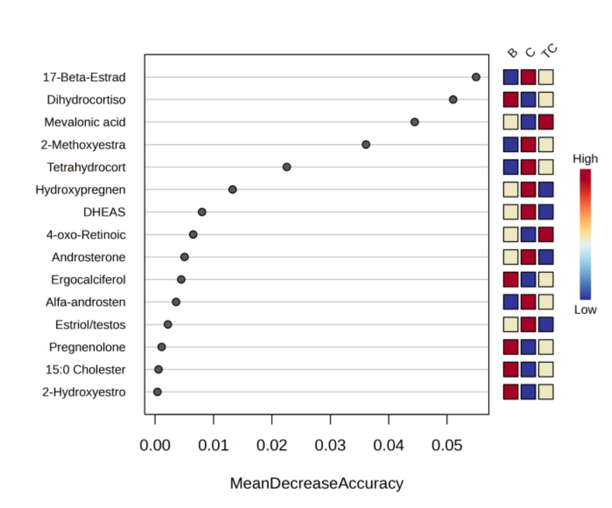


**i.** Steroids

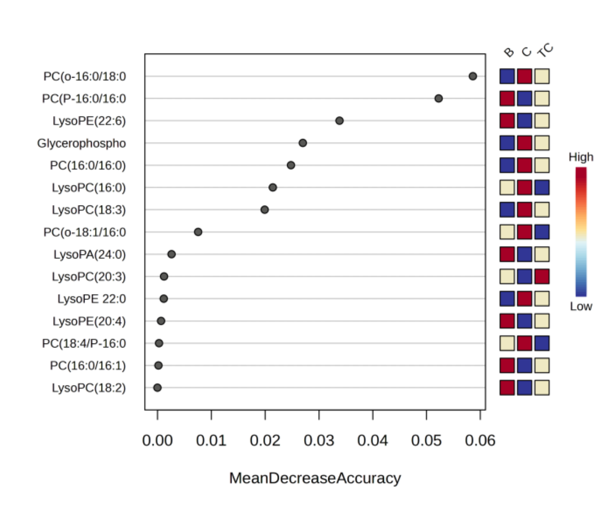


**j.** Phospholipids
