# Supplementary material for: In Search of Relevant Urinary Biomarkers for Thyroid Papillary Carcinoma and Benign Thyroid Nodule Differentiation, Targeting Metabolic Profiles and Pathways via UHPLC-QTOF-ESI+-MS Analysis
Source: Diagnostics (Basel). 2024 Oct 30;14(21):2421. doi: 10.3390/diagnostics14212421 (PMC11544950; doi:10.3390/diagnostics14212421)
Supplement: Supplementary file 1 [file diagnostics-14-02421-s001.zip › Supl File Table S1 Identifications.pdf]

Table S1. Urine molecules (n=190) separated using HPLC-QTOF-ESI$^+$-MS and identified, according to their m/z values. The experimental m/z values were compared with the average of theoretical m/z values from the International database HMDB (Human Metabolomic DataBase). The accuracy of (theoretical – experimental) m/z values was below 20 ppm. The ID HMDB codes are mentioned in column 3.

m/z	Identification	ID (HMDB)
**TCA metabolites and phenol derivatives (n=14)**		
109.1027	p-Cresol	HMDB0001858
111.1183	Hydroquinone	HMDB0002434
117.093	Fumaric acid	HMDB0000134
119.0877	Succinic acid	HMDB0000254
139.0204	3-Hydroxybenzoic acid	HMDB0002466
151.5860	Phenyl lactic acid	HMDB0000814
155.1465	Dihydroxybenzoic acid	HMDB29666
161.1203	Tryptamine	HMDB0000303
169.1117	Homogentisic acid	HMDB0000130
173.0275	Glycerol 3-phosphate	HMDB0000126
174.9959	Pyrophosphate	HMDB0000250
177.0599	N-Hydroxyl tryptamine	HMDB0255154
181.1253	Glucose	HMDB0000126
197.1576	Gluconic acid	HMDB0000625
**Amino acids, amines and metabolites (n=35)**		
116.109	L-Proline	HMDB0000162
125.9926	Taurine	HMDB0000251
131.109	Ketoleucine	HMDB0000695
132.1046	Isoleucine	HMDB0000172
134.0633	Aspartic acid	HMDB0000191
143.9996	Proline betaine	HMDB0004827
147.0221	Lysine	HMDB0000182
148.0827	Glutamic acid	HMDB0000148
156.0802	Histidine	HMDB0000177
158.1566	Tiglylglycine	HMDB0000959
160.1835	N-Acetylproline	HMDB0094701
163.0777	3-Hydroxymethylglutaric acid	HMDB0000355
163.647	5-hydroxylysine	HMDB0000450
170.028	1-Methylhistidine	HMDB0000001
173.1574	Glycylproline	HMDB0000721
175.136	Argininic acid	HMDB0003148
176.0426	Tyrosine	HMDB0000158
177.1664	Serotonin	HMDB0000259
186.0823	DL-O-Phosphoserine	HMDB0001721
188.5820	N1-Acetylspermidine	HMDB0001276
200.2414	O-Phosphothreonine	HMDB0011185
203.1832	Spermine	HMDB0001256
205.0995	Tryptophan	HMDB0000684
213.1822	Acetyl hydroxytryptamine	HMDB0001238
214.2576	Indoxyl sulfate	HMDB0000682
221.1395	5 Hydroxy tryptophan	HMDB0000471
223.1017	L-Cystathionine	HMDB0000099

232.5734	Suberylglycine	HMDB0000953
233.1952	Melatonin	HMDB01389
247.2327	N-acetyltryptophan	HMDB0013713
249.2120	6-Hydroxymelatonin	HMDB0004081
265.1672	di-Hydroxymelatonin	HMDB0061136
308.2886	Glutathionate	HMDB0062697
314.3518	Palmitoylglycine	HMDB0013034
394.3667	N acetyl serotonin glucuronide	HMDB60833
**Butyric derivatives, Methionine& Cysteine Selenocomplexes (n=10)**		
103.0403	2-Ketobutyric acid	HMDB0000005
105.035	3-Hydroxybutyric acid	HMDB0000357
121.103	(S)-3,4-Dihydroxybutyric acid	HMDB0000337
136.0338	Homocysteine	HMDB0000742
150.1149	Methionine	HMDB0000696
164.033	Acetylcysteine	HMDB0001890
172.1007	L-Homocysteine sulfate	HMDB0002238
183.1683	Methylselenocysteine	HMDB0004113
196.918	Selenomethionine	HMDB0003966
202.2204	Cysteine-S-sulfate	HMDB0000731
**Purines and pirimidine derivatives (n=27)**		
114.0281	Creatinina	HMDB0000562
115.1136	Dihydrouracil	HMDB0000076
127.0954	Thymine	HMDB0000262
128.9876	2-Thiouracil	HMDB0245323
129.1295	Dihydrothymine	HMDB0000079
152.1115	Hydroxy adenine	HMDB0000403
153.1298	Oxypurinol	HMDB0000786
157.0851	Orotic acid	HMDB0000226
167.1095	3-Methylxanthine	HMDB0001886
168.0404	Thioguanine	HMDB0014496
171.1521	Propylthiouracil	HMDB0014690
180.1395	Hippuric acid	HMDB0000714
181.5583	Nicotin uric acid	HMDB0003269
185.1217	6-thiouric acid	HMDB0060417
211.2104	1,3,7-Trimethyluric acid	HMDB0002123
228.2739	Deoxycytidine	HMDB0000014
243.1637	Thymidine	HMDB0000273
245.0849	Uridine	HMDB0000296
253.0018	Deoxyinosine	HMDB0000071
253.1846	Deoxyadenosine	HMDB0000101
269.2104	Inosine	HMDB0000195
284.336	Guanosine	HMDB0000133
298.2136	1-Methylguanosine	HMDB0001563
312.3682	N2,N2-Dimethylguanosine	HMDB0004824
325.258	Uridine 5'-monophosphate	HMDB0000288
348.997	Adenosine monophosphate	HMDB0000045
399.3159	S-Adenosylmethionine	HMDB0001185
**Lipids: Fatty acids, sphingolipids and ceramides (n=17)**		
145.0190	Caprylic acid	HMDB0000482

229.146	Myristic acid C14:0	HMDB0000806
257.2524	Palmitic acid C16:0	HMDB0000220
279.2371	Linolenic acid C18:3	HMDB0003073
283.2651	Oleic acid C18:1	HMDB0000207
285.2686	Stearic acid C18:0	HMDB0000827
300.2616	Sphingosine	HMDB0000252
307.261	Eicosatrienoic acid C20:3	HMDB0002925
311.2621	Eicosenoic acid C20:1	HMDB0002231
313.2395	Arachidic acid C20:0	HMDB0002212
316.3279	Dehydrophytosphingosine	HMDB0038057
321.2517	15(S)-HETE	HMDB0003876
329.2568	Docosahexenoic acid C22:6	HMDB0002183
540.4447	Ceramide(d18:0/16:0)	HMDB0011760
704.5512	SM(d18:0/16:1)	HMDB0013464
707.5365	SM(d18:0/16:0)	HMDB0010168
730.6052	SM(d18:1/18:1)	HMDB0012100
**AcylCarnitines (n=22)**		
162.1152	L-carnitine	HMDB0000062
216.2365	Propenoylcarnitine	HMDB0000200
230.2524	Butenylcarnitine	HMDB0013126
244.2682	Tiglylcarnitine	HMDB0002366
248.0989	Malonylcarnitine	HMDB0002095
274.2799	Glutaconylcarnitine	HMDB0013129
286.3161	Octenoylcarnitine	HMDB0013324
288.2957	Octanoylcarnitine	HMDB0000791
290.2771	Adipoyl carnitine	HMDB0061677
302.2412	Nonanoyl carnitine	HMDB0013288
304.3065	3-hydroxydecanoyl carnitine	HMDB0061636
314.3518	Decenoylcarnitine	HMDB0241072
332.3381	Tetradecanoylcarnitine	HMDB0005066
342.3798	9,12-Hexadecadienoylcarnitine	HMDB0013334
342.3848	9,12-Hexadecadienoylcarnitine	HMDB0013334
344.3272	Dodecanoyl carnitine	HMDB0000944
372.3551	trans-Hexadec-2-enoyl carnitine	HMDB0006317
400.3867	Methyldocosanoylcarnitine (adduct)	HMDB0240952
414.3375	Heptadecanoyl carnitine	HMDB0006210
424.4435	Linoleoyl carnitine	HMDB0006681
428.3246	Arachidyl carnitine	HMDB0006460
472.3326	Cervonyl carnitine	HMDB0006510
**Bile acids (n=9)**		
227.1801	Porphobilinogen	HMDB0000250
377.226	Lithocholic acid	HMDB0000717
391.299	12-Ketodeoxycholic acid	HMDB0000328
393.32	7-Ketodeoxycholic acid	HMDB0000502
423.331	Ursocholic acid	HMDB0000917
484.393	Chenodeoxycholic acid	HMDB0000518
500.389	Deoxycholic acid	HMDB0000626
516.362	Taurocholic acid	HMDB0000036
553.401	Lithocholic acid glucuronide	HMDB0002513

**Steroids and vitamins (n=26)**		
149.0725	Mevalonic acid	HMDB0000227
266.2390	Thiamine	HMDB0000235
271.1949	Estrone	HMDB0000145
275.2651	Alfa-androstenol	HMDB0006571
277.1880	19-norandrosterone	HMDB0002697
287.1863	2-Hydroxyestrone	HMDB0000343
289.1591	Estriol	HMDB0000151
291.2608	Androsterone	HMDB0000031
301.1502	2-Methoxyestrone	HMDB0000010
303.2615	2-Methoxyestradiol-17beta	HMDB0000405
305.1796	4-Hydroxytestosterone	HMDB0246468
315.2653	4-oxo-Retinoic acid	HMDB0006285
317.2197	Pregnenolone	HMDB0000253
333.2891	16-a-Hydroxypregnenolone	HMDB0000315
361.2327	Cortisone	HMDB0002802
363.2255	Cortisol	HMDB0000063
365.1439	Tetrahydrocortisone	HMDB0000903
365.2855	Dihydrocortisol	HMDB0003259
369.2571	Dehydroepiandrosterone 3-sulfate (DHEAS)	HMDB0001032
387.1046	Cholesterol	HMDB0000067
397.3067	Ergocalciferol	HMDB0000161
401.2837	5,6-trans-25-Hydroxyvitamin D3	HMDB0006721
433.2733	17-Beta-Estradiol-3,17-beta-sulfate	HMDB0041620
443.3486	Cortisol 21- sulfate	HMDB62779
611.3941	15:0 Cholesterol ester	HMDB0060057
653.4270	18:0 Cholesterol ester	HMDB0010368
**Prostaglandins (n=6)**		
335.1374	PGA2/B2	HMDB0002752
337.2411	PGA1	HMDB0002656
353.2739	PGE2 /D2	HMDB0060041
355.2895	PGF2a	HMDB0001139
357.2869	PGF1a	HMDB0002685
381.3058	Dimethyl-PGE2	HMDB0244730
**Phospholipids (n=24)**		
258.2841	Glycerophosphocholine	HMDB0000086
435.2664	LysoPA(18:2)	HMDB0007856
496.3725	LysoPC(16:0)	HMDB0010382
502.3828	LysoPE(20:4)	HMDB0011487
518.3312	LysoPC(18:3)	HMDB0010387
520.3492	LysoPC(18:2)	HMDB0010386
523.3199	LysoPA(24:0)	HMDB0114756
526.4563	LysoPE(22:6)	HMDB0011496
528.4369	LysoPE(22:5)	HMDB0011494
539.3830	LysoPE 22:0	HMDB11520
542.3328	LysoPC(20:5)	HMDB0010397
546.387	LysoPC(20:3)	HMDB0010393
570.4166	LysoPC(22:5)	HMDB0010402

572.4492	LysoPC(22:4)	HMDB0010401
717.5136	PC(P-16:0/16:1)	HMDB0011207
719.2902	PC(P-16:0/16:0)	HMDB0011206
732.5668	PC(16:0/16:1)	HMDB0007969
735.5372	PC(16:0/16:0)	HMDB0000564
738.4135	PC(18:4/P-16:0)	HMDB0008258
741.4901	PC(18:3/P-16:0)	HMDB0008225
743.4930	PC(18:2/P-16:0)	HMDB0008159
745.5648	PC(18:1/P-16:0)	HMDB0008093
746.6098	PC(O-18:1/16:0)	HMDB0013426
748.5922	PC(O-16:0/18:0)	HMDB0013405
