# Supplementary material for: In Search of Relevant Urinary Biomarkers for Thyroid Papillary Carcinoma and Benign Thyroid Nodule Differentiation, Targeting Metabolic Profiles and Pathways via UHPLC-QTOF-ESI+-MS Analysis
Source: Diagnostics (Basel). 2024 Oct 30;14(21):2421. doi: 10.3390/diagnostics14212421 (PMC11544950; doi:10.3390/diagnostics14212421)
Supplement: Supplementary file 1 [file diagnostics-14-02421-s001.zip › Supl file Table S2 common and specific molecules.pdf]

Table S2. Common and specific molecules identified in urine comparative to blood serum, as determined by Venny 2.1 algorithm.

**Common molecules (90)**	**Specific molecules in blood (76)**	**Specific molecules in urine (100)**
12-Ketodeoxycholic acid	(S)-3,4-Dihydroxybutyric acid	15(S)-HETE
1-Methylguanosine	1-Methylhistidine	15:0 Cholesterol ester
2-Thiouracil	3-Methyluridine	17-Beta-Estradiol sulfate
3-hydroxydecanoyl carnitine	3-Oxocholic acid	18:0 Cholesterol ester
3-Methylxanthine	5-Hydroxy-L-tryptophan	19-Norandrosterone
3-OH butyric acid	5-hydroxylysine	25-Hydroxyvitamin D3
7-Ketodeoxycholic acid	5-Hydroxymethyluracil	2-Hydroxyestrone
9-Decenoylcarnitine	7-Methyl-cholic acid	2-Ketobutyric acid
Acetyl hydroxytryptamine	9-Hexadecenoylcarnitine	2-Methoxyestradiol
Acetylcysteine	Adenine	2-Methoxyestrone
Adenosine monophosphate	Adenosine	3,4-DiOHbutyric acid
Arachidyl carnitine	Arachidic acid C20:0	3-OHbenzoic acid
Arginine	Arahidonic acid C20:4	3-OHmethylglutaric acid
Butenylcarnitine	Argininic acid	4-Hydroxytestosterone
Cervonyl carnitine	Asparagine	4-oxo-Retinoic acid
Chenodeoxycholic acid	Citric acid	5 Hydroxy lysine
Cortisol	D-2-Hydroxyglutaric acid	5-OH tryptophan
Cortisone	Deoxycholic acid glycine conjugate	6-Hydroxymelatonin
Cysteine-S-sulfate	Deoxycortisol	6-thiouric acid
Deoxyadenosine	Dinor-6-keto-PGF1a	Adipoyl carnitine
Deoxycholic acid	Docosahexenoic acid C22:6	Alfa-androstenol
Deoxyinosine	Eicosadienoic acid C20:2	Androsterone
Dihydrocortisol	GABA	Arachidic acid
Dihydrothymine	Glutarylcarnitine	Aspartic acid
Dihydrouracil	Glycyl-Histidine	Caprylic acid
Dimethyl-PGE2	Guanosine monophosphate	Ceramide(d18:0/16:0)
Dodecenoylcarnitine	Hydroxycortisol	Cholesterol
Eicosatrienoic acid C20:3	Hypotaurine	Cortisol 21- sulfate
Eicosenoic acid C20:1	Hypoxanthine	Creatinine
Fumaric acid	Inosinic acid	Dehydrophytosphingosine
Glutaconylcarnitine	Isoleucine	Deoxycytidine
Glutamic acid	Kynurenine	DHEAS
Glycerophosphocholine	L-Acetylcarnitine	DiHydroxymelatonin
Glycylproline	L-Cystine	Dimethylguanosine
Guanosine	L-Glutamine	Di-OHbenzoic acid
Hexadecenoyl carnitine	Linoleic acid C18:2	Docosahexenoic acid
Histidine	Linolenic acid C18:3	Dodecanoyl carnitine
Homocysteine	Lithocholyltaurine	Ergocalciferol

Hydroxy adenine	L-malic acid	Estriol/testosterone
Inosine	L-Octanoylcarnitine	Estrone
Ketoleucine	L-Palmitoylcarnitine	Gluconic acid
L-carnitine	L-Tryptophan	Glucose
L-Homocysteine sulfate	LysoPA(18:0)	Glycerol 3-phosphate
Lithocholic acid	LysoPA(22:1)	Heptadecanoyl carnitine
Lithocholic acid glucuronide	LysoPC(16:1)	Hippuric acid
L-Proline	LysoPC(18:0)	Homogentisic acid
Lysine	LysoPC(18:1)	Hydroquinone
LysoPA(18:2)	LysoPC(20:4)	Hydroxypregnenolone
LysoPA(24:0)	LysoPC(22:6)	Indoxyl sulfate
LysoPC(16:0)	LysoPE(22:2)	L-Cystathionine
LysoPC(18:2)	Methyl arachidic acid C21:0	Leucine
LysoPC(18:3)	Methyldocosanoylcarnitine	Linolenic acid
LysoPC(20:3)	Myristic acid C14:0	Linoleoyl carnitine
LysoPC(20:5)	N2,N2-Dimethylguanosine	LysoPE 22:0
LysoPC(22:4)	N-Acetyl-L-tyrosine	Malonylcarnitine
LysoPC(22:5)	Norcholic acid	Melatonin
LysoPE(20:4)	Oleic acid C18:1	Methyl arachidic acid
LysoPE(22:5)	Oxocortisol	Methylhistidine
LysoPE(22:6)	Oxoproline	Mevalonic acid
Methionine	Palmitic acid C16:0	Myristic acid
Methylselenocysteine	Palmitoleic acid C16:1	N-acetyl serotonin glucuronide
N-Acetylproline	PGE3/D3	N-Acetylspermidine
Octenoylcarnitine	Phenylalanine	N-acetyltryptophan
PC(16:0/16:1)	Pimelylcarnitine	Nicotinuric acid
PGA1	Propionylcarnitine	Nonanoyl carnitine
PGA2/B2	Stearic acid C18:0	Octanoylcarnitine
PGE2 /D2	Stearoylcarnitine	Oleic acid
PGF1a	Taurodeoxycholic acid	O-Phosphoserine
PGF2a	Tetrahydrocortisol	O-Phosphothreonine
Proline betaine	Threonine	Orotic acid
Propenoylcarnitine	Tyramine	Oxypurinol
Propylthiouracil	Uracil	Palmitic acid
S-Adenosylmethionine	Uridine 5'-diphosphate	Palmitoyl carnitine
Selenomethionine	Valerylcarnitine	PC(16:0/16:0)
Serotonin	Valine	PC(18:1/P-16:0)
Succinic acid	Xanthosine	PC(18:2/P-16:0)
Taurine		PC(18:3/P-16:0)
Taurocholic acid		PC(18:4/P-16:0)
Tetradecanoylcarnitine		PC(O-16:0/18:0)

Tetrahydrocortisone		PC(O-18:1/16:0)
Thymidine		PC(P-16:0/16:0)
Thymine		PC(P-16:0/16:1)
Tiglylcarnitine		p-Cresol
Tryptamine		Phenyl lactic acid
Tyrosine		Porphobilinogen
Uridine 5'-monophosphate		Pregnenolone
Ursocholic acid		Pyrophosphate
Hexadecadienoylcarnitine		SM(d18:0/16:0)
5-Hydroxytryptamine		SM(d18:0/16:1)
Glutathione		SM(d18:1/18:1)
		Spermine
		Sphingosine
		Stearic acid
		Suberylglycine
		Thiamine
		Thioguanine
		Tiglylglycine
		Trimethyluric acid
		Tryptophan
		Uridine
